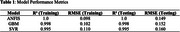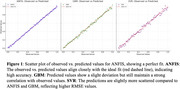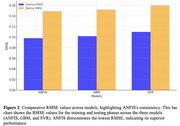# Comparative evaluation of machine learning models for dementia prediction

**DOI:** 10.1002/alz70856_098569

**Published:** 2025-12-24

**Authors:** Efe Precious Onakpojeruo, Dilber Uzun Ozsahin, Berna Uzun, Ilker Ozsahin

**Affiliations:** ^1^ Operational Research Center in Healthcare, Near East University, Nicosia/TRNC, Mersin 10, Turkey; ^2^ Medical Diagnostic Imaging Department, College of Health Sciences, University of Sharjah, 27272, Sharjah, United Arab Emirates

## Abstract

**Background:**

Dementia remains a global health challenge, with over 50 million affected individuals and projections suggesting a tripling by 2050. Early and accurate prediction is critical for effective management. The aim of this study is to explore advanced machine learning techniques to enhance predictive accuracy.

**Method:**

The predictive capabilities of three machine learning models; Support Vector Regression (SVR), Gradient Boosting Machine (GBM), and Adaptive Neuro‐Fuzzy Inference System (ANFIS) in the context of dementia diagnosis were investigated. A dataset of 149 participants aged 60–96 years, incorporating nine clinical and imaging biomarkers, was utilized for model training and validation splits of 70% and 30%, respectively. Key evaluation metrics included R^2^, RMSE, and MSE.

**Result:**

The results demonstrated that all models accurately predicted dementia, with ANFIS outperforming SVR and GBM in terms of precision and consistency. ANFIS achieved an R^2^ of 1.0 during both training and testing phases, while GBM and SVR also displayed high accuracy with R^2^ values of 0.998 and 0.995, respectively. The results validate the efficacy of hybrid and ensemble approaches like ANFIS for dementia prediction.

**Conclusion:**

These findings underscore the potential of ensemble and hybrid machine learning approaches in enhancing dementia prediction accuracy, paving the way for early and reliable diagnosis.